# Prodromal Parkinsonism and Neurodegenerative Risk Stratification in REM Sleep Behavior Disorder

**DOI:** 10.1093/sleep/zsx071

**Published:** 2017-05-04

**Authors:** Thomas R Barber, Michael Lawton, Michal Rolinski, Samuel Evetts, Fahd Baig, Claudio Ruffmann, Aimie Gornall, Johannes C Klein, Christine Lo, Gary Dennis, Oliver Bandmann, Timothy Quinnell, Zenobia Zaiwalla, Yoav Ben-Shlomo, Michele TM Hu

**Affiliations:** 1 Oxford Parkinson’s Disease Centre (OPDC), University of Oxford, UK;; 2 Nuffield Department of Clinical Neurosciences, University of Oxford, UK;; 3 School of Social and Community Medicine, University of Bristol, UK;; 4 Institute of Clinical Neurosciences, University of Bristol, UK;; 5 Department of Psychiatry, University of Oxford, UK;; 6 Sheffield Institute of Translational Neuroscience, University of Sheffield, UK;; 7 Department of Neurology, Sheffield Teaching Hospitals, Sheffield, UK;; 8 Respiratory Support and Sleep Centre, Papworth Hospital, Cambridge, UK;; 9 Department of Clinical Neurophysiology, John Radcliffe Hospital, Oxford, UK

**Keywords:** RBD, Prodromal, Neurodegeneration, Parkinson’s Disease

## Abstract

**Objectives:**

Rapid eye movement (REM) sleep behavior disorder (RBD) is the most specific marker of prodromal alpha-synucleinopathies. We sought to delineate the baseline clinical characteristics of RBD and evaluate risk stratification models.

**Methods:**

Clinical assessments were performed in 171 RBD, 296 control, and 119 untreated Parkinson’s (PD) participants. Putative risk measures were assessed as predictors of prodromal neurodegeneration, and Movement Disorders Society (MDS) criteria for prodromal PD were applied. Participants were screened for common leucine-rich repeat kinase 2 (LRRK2)/glucocerebrosidase gene (GBA) gene mutations.

**Results:**

Compared to controls, participants with RBD had higher rates of solvent exposure, head injury, smoking, obesity, and antidepressant use. GBA mutations were more common in RBD, but no LRRK2 mutations were found. RBD participants performed significantly worse than controls on Unified Parkinson’s Disease Rating Scale (UPDRS)-III, timed “get-up-and-go”, Flamingo test, Sniffin Sticks, and cognitive tests and had worse measures of constipation, quality of life (QOL), and orthostatic hypotension. For all these measures except UPDRS-III, RBD and PD participants were equally impaired. Depression, anxiety, and apathy were worse in RBD compared to PD participants. Stratification of people with RBD according to antidepressant use, obesity, and age altered the odds ratio (OR) of hyposmia compared to controls from 3.4 to 45.5. 74% (95% confidence interval [CI] 66%, 80%) of RBD participants met the MDS criteria for probable prodromal Parkinson’s compared to 0.3% (95% CI 0.009%, 2%) of controls.

**Conclusions:**

RBD are impaired across a range of clinical measures consistent with prodromal PD and suggestive of a more severe nonmotor subtype. Clinical risk stratification has the potential to select higher risk patients for neuroprotective interventions.

**Statement of Significance**

This is the largest study to date comparing the clinical characteristics of rapid eye movement (REM) sleep behavior disorder (RBD) patients with Parkinson’s disease (PD) patients and healthy controls. Our data show that people with RBD have a nonmotor phenotype that is as severe as that seen in early PD, suggesting that they represent the prodromal phase of a worse nonmotor disease subtype. We found that antidepressant use and obesity are common in RBD and associated with a lower probability of hyposmia, perhaps indicating a lower near-term conversion risk. We have also evaluated the new Movement Disorder Society Research Criteria for Prodromal Parkinson’s and identified some important strengths and limitations. Longitudinal follow-up will determine the true predictive value of these risk models.

## INTRODUCTION

Emerging evidence over the past 15 years has established rapid eye movement (REM) sleep behavior disorder (RBD) as a highly specific marker of the prodromal phase of alpha-synucleinopathies, in particular Parkinson’s disease (PD), dementia with Lewy bodies (DLB), and multiple system atrophy (MSA).^1^ This parasomnia, characterized by the loss of normal muscle atonia during REM sleep, is associated with a future risk of neurodegenerative disease reaching more than 80% in some studies.^2^ There is, however, considerable variation in these conversion rates among different cohorts worldwide, and even within cohorts, the latency to onset of a defined neurodegenerative disorder is highly variable.^3–5^

Accurate identification of those patients at highest risk of imminent phenoconversion would facilitate recruitment to trials of neuroprotective agents aimed at delaying the onset of alpha-synucleinopathies.^6^ Given the large numbers of patients needed for such trials, risk stratification methods must be standardized and reproducible across different geographical regions. One method of risk stratification recently proposed by Berg et al.^7^ is the Movement Disorder Society (MDS) Criteria for Prodromal PD.^7^ This method takes the likelihood ratios for future PD conferred by a number of background risk factors and early neurodegenerative signs and combines them into a probability score, with a suggested threshold of 80% indicating probable prodromal PD. Application of these criteria in population and prodromal cohorts has yielded promising results,^8,9^ but they require further validation in prospective cohort studies.

Part of the variation in latency from RBD diagnosis to conversion may be a result of differences in the time at which patients present to sleep services, such that patients presenting later may be at a more advanced prodromal stage. We sought to explore the effect of common comorbidities that may influence this. One such comorbidity is depression because the use of antidepressants can exacerbate RBD symptoms and may therefore unmask the condition at an earlier stage.^10^ Respiratory sleep disorders may conceivably exert a similar effect. Concomitant obstructive sleep apnea (OSA) affects 34%–61% of patients with RBD,^11–13^ and it is common for RBD to be diagnosed following an initial referral for suspected OSA.^14^ Given the link between body mass index (BMI) and sleep-disordered breathing, we hypothesized that individuals with high BMI may also present at an earlier neurodegenerative stage.

Another factor that may contribute to regional variations among RBD cohorts is genetic risk. Recent evidence suggests that the genetic profile of RBD is not the same as for PD in general. Mutations in the leucine-rich repeat kinase 2 (LRRK2) gene are the most common cause of familial PD,^15^ but patients with LRRK2-associated PD have lower rates of RBD than seen in sporadic PD,^16^ and LRRK2 mutations were not detected in a large Spanish cohort of idiopathic RBD.^17^ Mutations in the glucocerebrosidase gene (GBA), on the other hand, appear to be associated with a more severe nonmotor phenotype in established PD^18,19^ and have a high prevalence in idiopathic RBD.^20^ The rates of such genetic risk factors show substantial variation among different ethnic groups,^21^ and these findings therefore require validation in geographically distinct populations.

Here, in the largest study of its kind to date, we comprehensively assess the baseline clinical, genetic, and background characteristics of a UK cohort of 171 patients with idiopathic RBD, comparing them with 296 control participants and 119 patients with early PD. We use these clinical characteristics to explore models that may stratify neurodegenerative risk.

## METHODS

### Subjects

Patients with idiopathic RBD were recruited from sleep disorders clinics at three centers: John Radcliffe Hospital, Oxford; Papworth Hospital, Cambridge; and Sheffield Teaching Hospital. The diagnosis of RBD was made on the basis of polysomnographic (PSG) evidence according to International Classification of Sleep Disorders criteria.^22^ Individuals with concomitant OSA were only included if the two conditions were unequivocally distinguishable by PSG. In uncertain cases, the diagnosis of RBD was either confirmed by repeat PSG with the use of continuous positive airway pressure, or the individuals were excluded from the study.

Healthy controls and patients with PD (diagnosed according to UK PD Brain Bank Criteria^23^) were selected from the Discovery Cohort of the Oxford Parkinson’s Disease Centre, a community ascertained cohort recruited from the Thames Valley region. In order to avoid the potential confounding effects of antiparkinsonian medication and to establish a comparison with the PD population closest to the prodromal phase, we only included patients with early, untreated PD. Full details of our clinical protocol are described elsewhere.^24^ The study was approved by the local research ethics committee and informed, written consent was given by all participants.

### Subject Evaluation

A comprehensive, structured medical history was taken from all participants including comorbidities, demographic information, environmental and occupational exposures, medications, and family history. Motor features were assessed using: part III of the MDS revised Unified Parkinson’s Disease Rating Scale (UPDRS^25^); the Purdue Pegboard test^26^; the Flamingo test (the ability of the patient to balance on one leg for 30 seconds), and the timed “get-up-and-go” test.^24^ Olfaction was assessed using the “Sniffin’ Sticks” odor identification test.^27^ Cognition was assessed using the Mini-Mental State Examination (MMSE^28^) and the Montreal Cognitive Assessment (MoCA^29^), with scores for the latter adjusted for years of education. Mild cognitive impairment was defined according to the MoCA diagnostic cutoff (<24/30).^24^ For phonemic fluency, the total number of words generated beginning with F, A, and S over 60 seconds was recorded. For semantic fluency, the number of animals and boys’ names each generated in 60 seconds was counted. Both fluency scores were adjusted for age. The Beck Depression Inventory (BDI-II^30^) and the Leeds Anxiety and Depression Scale (LADS^31^) were used to evaluate depression and anxiety respectively. Self-reported nonmotor symptoms were assessed using UPDRS part I. EQ-5D was used as a standardized self-report measure of health status.^32^ Cardiovascular risk factors were defined as: history of cardiovascular disease (angina, myocardial infarction, stroke, or transient ischemic attack); current smoker; hypertension; hypercholesterolemia; obesity (BMI > 30 kg/m^2^); and diabetes.

### Genetic Testing

Participants were screened for G2019S and R1441C mutations in the LRRK2 gene and N370S and L444P mutations in the GBA gene. For the LRRK2 screening, results were available for 289 controls, 136 patients with RBD, and 114 patients with PD. For the GBA screening, results were available for 283 controls, 116 RBD, and 106 participants with PD. DNA was extracted from whole blood using a Qiagen Autopure automated system. Polymerase chain reaction (PCR) was performed using MegaMix Blue (Microzone) containing a recombinant *Taq* polymerase. Primer sequences were as follows: G2019S: 5′-TTTAAGGGACAAA GTGAGCAC-3′ and 5′-ACTCTGTTTTCCTTTTGACTC-3′; R1441C: 5′-AAGGCATGAAGATGGGAAAG-3′ and 5′-TGA TGGTTTTCCGAAGTTTTG-3′; N370S: 5′-GCCTTTGTCCTT ACCCTC*G-3′ and 5′-GACAAAGTTACGCACCCAA-3′; L444P: 5′-GGAGGACCCAATTGGGTGCGT-3′ and 5′-ACG CTGTCTTCAGCCCACTTC-3′ (* indicates a mismatch that was introduced into the forward primer to create a restriction site).The PCR products for G2019S, R1441C, N370S, and L444P were digested with SfcI (BfmI), BstUI, XhoI, and NciI (BcnI), respectively and resolved by agarose gel electrophoresis.

### Statistical Analysis

Between-groups comparisons of clinical features were made using a linear regression model for continuous variables and a logistic regression model for dichotomous variables. As the groups were not precisely age and gender matched, we included age and gender as covariates in the model to control for any effect of these (except in the case of genetic data, where we included gender but not age). Statistical significance is presented as absolute *p* values, uncorrected for multiple comparisons. Borderline *p* values should therefore be interpreted with caution due to the possibility of a type I error. A sensitivity analysis was performed excluding the 12 RBD participants who had uncorrected moderate or severe OSA at the time of their diagnostic PSG.

### Risk Stratification of RBD Versus Controls

We selected three risk factors that may help identify RBD cases where imminent phenoconversion to a neurodegenerative disorder is more likely. These were as follows: nonuse (high risk) versus use (low risk) of antidepressants; presence (low risk) or absence (high risk) of obesity (BMI > 30kg/m^2^); and age above (high risk) versus below (low risk) 60 years. We then looked at the ability of these measures to predict the presence of hyposmia (Sniffin Sticks score < 10th centile adjusted for age and gender^23^), a surrogate marker of early neurodegeneration. Hyposmia was selected as the outcome because it is a common, early prodromal symptom (corresponding to stage 1 of the Braak hypothesis^33^) and, unlike motor or cognitive performance, it is unlikely to be influenced by obesity or depression themselves. To compare how each risk factor modified the overall risk of hyposmia between RBD cases and controls, we ran a series of multivariable logistic regression models to derive the OR, with interaction terms for each risk factor and case status (RBD vs. control).

### MDS Criteria for Prodromal PD

The probability of prodromal PD was calculated for each participant at their baseline assessment using the method described by Berg et al.^7^ We used the following risk markers: sex, pesticide exposure, solvent exposure, caffeine use, smoking history, family history of PD, and presence of gene mutation (GBA or LRRK2). The following prodromal markers were available for inclusion: RBD screening questionnaire (RBDSQ^34^), subthreshold parkinsonism (using UPDRS and Purdue Pegboard scores), olfactory loss, constipation, excessive daytime somnolence (measured by the Epworth Sleepiness Scale^35^), postural hypotension, urinary dysfunction, and depression/anxiety. PSG-proven RBD data were available for the RBD participants only, where it was used instead of the RBDSQ. The likelihood ratio for motor impairment was included in the calculation for all participants, including those with Parkinson’s. In cases where data were missing or ambiguous, a likelihood ratio of 1 was used.

## RESULTS

A total of 171 patients with idiopathic RBD (mean symptom duration 7.07 years, standard deviation [SD] 6.30), 296 healthy controls, and 119 participants with early, untreated PD (mean time since diagnosis 0.78 years, SD 0.78) were included in the study.

### Demographics and Background Risk Factors

Key demographic, environmental, and genetic risk factors are shown in Table 1. Compared to controls, RBD participants had significantly higher rates of self-reported head injury and solvent exposure, both known risk factors for PD. Obesity and smoking were significantly more common in RBD participants than controls or patients with PD, and the total number of cardiovascular risk factors was also higher in patients with RBD. The RBD group had a significantly higher rate of antidepressant use than controls or patients with PD and a significantly shorter duration of formal education.

**Table 1:** Demographics and Background Risk Factors.

Demographic/background risk variable	Controls ***N*** = 296	RBD ***N*** = 171	PD ***N*** = 119	***p*** value	
Age, mean (SD)	64.9 (10.2)	64.7 (9.0)	66.9 (9.1)	RBD versus control: RBD versus PD: PD versus control:	.79 .06 .06
Sex (% male)	49.0	88.3	70.6	RBD versus control: RBD versus PD: PD versus control:	**<.001** **<.001** **<.001**
Body mass index (BMI) Mean, kg/m^2^ (SD)	27.4 (4.93)	29.1 (5.91)	26.1 (3.73)	RBD versus control: RBD versus PD: PD versus control:	**.003** **<.001** **.02**
Pesticide exposure* (%)	37.2	43.9	33.9	RBD versus control: RBD versus PD: PD versus control:	.27 .10 .44
Solvent exposure+ (%)	11.1	22.2	18.6	RBD versus control: RBD versus PD: PD versus control:	**.05** .93 .10
Caffeine intake**	4.71 (2.24)	5.39 (2.70)	5.00 (2.87)	RBD versus control: RBD versus PD: PD versus control:	.08 .41 .47
Head injury++ (%)	18.2	32.2	22.9	RBD versus control: RBD versus PD: PD versus control:	**.02** .24 .41
Smoking history*** (%)	43.2	63.2	40.2	RBD versus control: RBD versus PD: PD versus control:	**.002** **<.001** .31
Obesity^ (%)	23.3	36.8	16.0	RBD versus control: RBD versus PD: PD versus control:	**.002** **<.001** .16
Education (years)	15.1 (3.45)	13.7 (3.39)	15.1 (3.86)	RBD versus control: RBD versus PD: PD versus control:	**<.001** **<.001** .64
Antidepressant use (%)	11.5	32.2	12.6	RBD versus control: RBD versus PD: PD versus control:	**<.001** **<.001** .25
GBA mutation^^ (%)	0.40	2.6	0.90	RBD versus control: RBD versus PD: PD versus control:	**.05** .31 .40
LRRK2 mutation^^ (%)	0	0	0.90	RBD versus control: RBD versus PD: PD versus control:	n/a 1.0 .99
Total number of cardiovascular risk factors, mean (SD)+++	1.01 (1.11)	1.55 (1.44)	1.08 (1.14)	RBD versus control: RBD versus PD: PD versus control:	**<.001** **<.001** .98

RBD participants have higher rates of chemical solvent exposure, head injury, smoking, obesity, and antidepressant use than controls. Shorter educational experience and higher total number of cardiovascular risk factors are also associated with RBD.

*Exposure to pesticides at work or home; +exposure to chemical solvents for > 6 months; **past caffeine intake: number of caffeinated drinks per day; ++history of head injury causing loss of consciousness or concussion diagnosed by a doctor; ***past or current smoking history; ^BMI > 30 kg/m^2^; +++risk factors defined as: history of cardiovascular disease (angina, myocardial infarction, stroke or transient ischaemic attack), diabetes, obesity, hypertension, current smoker, hypercholesterolaemia. For all variables except age, sex and GBA/LRRK2 status, *p* values for between groups comparisons are corrected for age and sex. The comparison of GBA/LRRK2 status is adjusted for sex only. ^^for the numbers of patients tested for genetic mutations, see main text.

GBA = glucocerebrosidase; LRRK2 = leucine-rich repeat kinase 2; RBD = rapid eye movement sleep behavior disorder; PD Parkinson’s disease; SD = standard deviation.

### Genetic Risk Variants

GBA mutations were detected in 3 out of 116 (2.6%) RBD participants for whom DNA analysis was available, compared to 1 out of 283 (0.4%) controls and 1 out of 106 (0.9%) PD. All GBA mutations detected were the N370S genotype. The difference in GBA mutation frequency between patients with RBD and controls was of borderline significance (*p* = .05). None of the 136 RBD or 289 control patients tested had either of the LRRK2 mutations G2019S or R1441C. One out of 114 patients with PD tested had the G2019S mutation.

### Early Motor Impairment in RBD

Table 2 summarizes the key motor and nonmotor features assessed in this study. Evidence of early motor impairment in RBD is demonstrated by significant differences between patients with RBD and controls in UPDRS-III scores, get-up-and-go times, and successful completion of the flamingo task. Although the UPDRS-III scores in patients with RBD reflected an intermediate phenotype between controls and patients with PD, patients with RBD and PD were equally impaired on the Flamingo and get-up-and-go tasks. There was little difference on the Purdue Pegboard test between RBD and control participants, who both performed better than PD cases.

**Table 2:** Motor and Nonmotor Features.

Clinical variable	Controls ***N*** = 296	RBD ***N*** = 171	PD ***N*** = 119	Significance (***p*** values)	
Non-motor	Values are mean (SD) unless otherwise stated				
UPDRS III, score	1.74 (2.74)	4.79 (5.97)	25.7 (11.1)	RBD versus controls: RBD versus PD: PD versus controls:	**<.001** **<.001** **<.001**
Purdue Pegboard, score	37.5 (6.80)	36.8 (8.04)	28.6 (6.49)	RBD versus controls: RBD versus PD: PD versus controls:	.69 **<.001** **<.001**
Flamingo, %	71.0	53.9	55.3	RBD versus controls: RBD versus PD: PD versus controls:	**<.001** .13 **.002**
Get up and go, time (seconds)	8.51 (1.73)	9.49 (3.17)	9.56 (2.24)	RBD versus controls: RBD versus PD: PD versus controls:	**<.001** .67 **<.001**
Nonmotor					
MMSE, score	28.3 (1.89)	27.3 (2.10)	27.6 (2.29)	RBD versus controls: RBD versus PD: PD versus controls:	**<.001** .13 **.002**
MoCA, score	26.7 (2.67)	25.1 (2.92)	25.2 (3.32)	RBD versus controls: RBD versus PD: PD versus controls:	**<.001** .64 **<.001**
Mild cognitive impairment (MoCA < 24) %	12.5	24.7	27.6	RBD versus versus controls: RBD versus PD: PD versus controls:	**.01** .57 **.003**
Semantic fluency, score	12.0 (3.33)	9.78 (3.35)	10.5 (3.34)	RBD versus controls: RBD versus PD: PD versus controls:	**<.001** .12 **<.001**
Phonemic fluency, score	12.8 (3.72)	10.4 (4.00)	11.5 (4.01)	RBD versus controls: RBD versus PD: PD versus controls:	**<.001** **.04** **.004**
Sniffin’ sticks, score	12.1 (2.28)	8.13 (3.26)	7.46 (2.88)	RBD versus controls: RBD versus PD: PD versus controls:	**<.001** .06 **<.001**
Orthostatic systolic blood pressure drop, mmHg	0.09 (12.3)	5.33 (13.6)	3.75 (13.5)	RBD versus controls: RBD versus PD: PD versus controls:	**<.001** .27 **.03**
Postural lightheadedness %	12.2*	42.3	27.1	RBD versus controls: RBD versus PD: PD versus controls:	**<.001** **.01** **.005**
Constipation, %	34.7	47.3	39.8	RBD versus controls: RBD versus PD: PD versus controls:	**.002** .08 .34
Beck Depression Inventory Score	4.85 (5.02)	10.25 (9.59)	7.55 (5.88)	RBD versus controls: RBD versus PD: PD versus controls:	**<.001** **.001** **<.001**
Leeds Anxiety Score	2.12 (2.38)	4.29 (3.84)	2.77 (2.98)	RBD versus controls: RBD versus PD: PD versus controls:	**<.001** **<.001** **.007**
Apathy (UPDRS part I, % > 0)	Not measured	29.8	16.2	RBD versus PD	**.02**
Quality of life EQ5D % score	84.5 (10.6)	74.0 (19.7)	76.0 (14.4)	RBD versus controls: RBD versus PD: PD versus controls:	**<.001** .28 **<.001**
UPDRS I, total score	Not measured	9.49 (6.45)	6.95 (4.53)	RBD versus PD	**<.001**

Patients with RBD are impaired in a wide range of motor and nonmotor characteristics compared to controls. In all measures except UPDRS III and Purdue Pegboard, participants with RBD are at least as impaired as those with early PD. In measures of depression, anxiety, apathy, phonemic fluency, and postural lightheadedness, people with RBD scored significantly worse than participants with PD.

All *p* values for two-way comparisons are corrected for age and gender differences between the groups.

*Data regarding this symptom was only available from 114 controls.

MMSE = Mini-Mental State Examination; MoCA = Montreal Cognitive Assessment; PD = Parkinson’s disease; RBD = rapid eye movement sleep behavior disorder; SD = standard deviation; UPDRS = Unified Parkinson’s Disease Rating Scale.

### Nonmotor Parkinsonian Features in RBD

Patients with RBD showed impairment in a wide range of parkinsonian nonmotor characteristics (Table 2). They performed significantly worse than controls in the MMSE, MoCA, semantic, and phonemic fluency tests, as well as in measures of olfaction, constipation, and orthostatic hypotension. In all of these tests, RBD participants were at least as impaired as PD participants. RBD participants were more likely to report symptoms of postural lightheadedness than either controls or people with PD.

Mood disorders were significantly worse in RBD participants than in controls or patients with PD. BDI and Leeds Anxiety Scale scores indicated a higher level of depression and anxiety in participants with RBD compared to controls or patients with PD, and participants with RBD were almost twice as likely as patients with PD to report apathy.

When asked to report their overall QOL using the EQ5D score, the reduction seen in RBD compared to control participants was as large as that seen in patients with established PD. Overall self-reporting of nonmotor symptoms in the UPDRS part I revealed significantly worse symptom scores in patients with RBD than in those with PD.

Table 3 shows the ORs for various parkinsonian features in patients with RBD compared to controls, adjusted for age and gender differences. As expected, patients with RBD showed increased ORs, varying from just over double (eg, cognitive impairment OR 2.04, 95% CI 1.19–3.49) to hyposmia which showed around a 14-fold relative odds (OR 13.8, 95% CI 8.13–23.4).

**Table 3:** Increased Risk of Parkinsonian Features in RBD Versus Controls.

Clinical feature	RBD versus controls adjusted for age and gender Odds ratio (95% CI)
Motor impairment^	6.46 (3.64–11.5)
Cognitive impairment*	2.04 (1.19–3.49)
Hyposmia**	13.8 (8.13–23.4)
Depression***	6.93 (3.54–13.5)
Anxiety^+^	6.45 (3.06–13.6)
Constipation^++^	2.07 (1.34–3.21)
Orthostatic hypotension^+++^	4.34 (2.12–8.88)

Patients with RBD have greatly increased risk of Parkinsonian features compared to controls.

^Unified Parkinson’s Disease Rating Scale (UPDRS) part III, score > 4; *Montreal Cognitive Assessment (MoCA) < 24; **Sniffin’ Sticks score < 10; ***Beck Depression Inventory score > 13; ^+^Leeds Anxiety Score > 6; ^++^less than one bowel movement per day or use of laxatives; ^+++^Orthostatic drop in systolic blood pressure > 20 mmHg.

CI = confidence interval; RBD = rapid eye movement sleep behavior disorder.

### The Effect of Antidepressants and Obesity

Within the group of patients with RBD, hyposmia was less severe in those taking antidepressant medication. Mean Sniffin Sticks score was 9.69 in those taking antidepressants compared to 7.36 in those not (*p* < .001). RBD participants taking antidepressants were also significantly younger (59.9 years vs. 67.0 years, *p* < .001). Importantly, these differences were not seen in the control group when comparing those taking and not taking antidepressants (*p* = .63 and *p* = .39 for the differences in Sniffin Sticks scores and age, respectively). A formal interaction test between patient group and antidepressant use revealed strong evidence of an interaction for both Sniffin Sticks (*p* < .001) and age (*p* = .022).

A similar effect on olfaction was seen relating to BMI. Patients with RBD who were not obese (BMI < 30 kg/m^2^) had significantly worse Sniffin Sticks scores (7.48 vs. 9.18, *p* = .001) than obese RBD participants. This effect was not seen in the control group (nonobese vs. obese Sniffin scores 12.0 vs. 12.1, *p* = .99), and a strong interaction was present between patient group and BMI for the Sniffin Sticks outcome (*p* = .003), suggesting that the effect of BMI on olfaction is specific to RBD.

The combination of participant status for obesity, antidepressant use, and age conferred an additive effect on the risk of impaired olfaction. Table 4 presents the ORs for hyposmia comparing patients with RBD with controls depending on the presence or absence of antidepressant use, obesity, and age. In patients with RBD, considered low risk for all three variables, the difference in risk compared to controls was consistent with chance (OR 3.39, 95% CI 0.78–14.8). Patients with RBD in the high risk category for all three variables had markedly increased risk (OR 45.5, 95% CI 21.1–98.0, *p* < .001).

**Table 4:** RBD Patient Stratification and Risk of Hyposmia.

Not on antidepressants	BMI < 30	Age > 60	Odds ratio (95% CI) for hyposmia*, RBD versus control
No	No	No	3.39 (0.78, 14.8)
Yes	No	No	5.63 (1.57, 20.2)
No	No	Yes	7.42 (1.91, 28.8)
Yes	No	Yes	12.3 (4.45, 34.0)
No	Yes	No	12.5 (2.62, 60.0)
Yes	Yes	No	20.8 (6.79, 63.8)
No	Yes	Yes	27.4 (6.56, 114.6)
Yes	Yes	Yes	45.5 (21.1, 98.0)

The odds ratio for hyposmia in RBD compared to controls increases more than 13-fold following risk stratification.

*Hyposmia defined as Sniffin Sticks score < 10th centile of normative data adjusted for age and gender.

BMI = body mass index; CI = confidence interval; RBD = rapid eye movement sleep behavior disorder.

### MDS Criteria for Prodromal Parkinson’s

Table 5 shows the probability of prodromal PD for the control, RBD, and early PD groups. Median values of absolute probability were 92.8% for patients with RBD, 0.48% for controls, and 52.2% for patients with early PD. Using the suggested MDS cutoff of more than 80% for a diagnosis of probable prodromal PD, around 74% (95% CI 66%–80%) of people with RBD fulfilled the criteria compared to 0.3% (95% CI 0.009%–2%) of controls and 21.8% (95% CI 14.8%–30.4%) of patients with early PD. With a lower threshold of more than 50%, 92.4% (95% CI 87.4%–95.6%) of patients with RBD met criteria compared to 1.4% (95% CI 0.4%–3.4%) of controls and 51.3% (95% CI 41.9%–60.5%) of patients with early PD.

**Table 5:** MDS Criteria for Prodromal Parkinson’s at Baseline According to PD, RBD, or Control Status and for RBD Converters to PD/DLB.

Probability value/threshold	Controls ***n*** = 296	RBD ***N*** = 171	PD ***N*** = 119	Converted from RBD to PD or DLB at follow-up (***N*** = 10)
Observed median probability of prodromal PD	0.48%	92.8%	52.2%	96.3%
> 80% probability	0.3%	73.7%	21.8%	80.0%
> 50% probability	1.40%	92.4%	51.3%	100%

Participants with RBD have a high probability of prodromal Parkinson’s according to MDS criteria. Only 0.3% of control participants fulfilled criteria using the suggested 80% cutoff. All the RBD participants who converted to PD had probability >50% at baseline.

All values are at baseline evaluation.

DLB = dementia with Lewy bodies; MDS = Movement Disorder Society; PD = Parkinson’s disease; RBD = rapid eye movement sleep behavior disorder.

### Significant Comorbid OSA

Twelve of the RBD participants had more than mild OSA that was uncorrected at PSG; by apnea-hypopnea index (AHI) this was classed as moderate (AHI 15–30) in 11 cases and severe (AHI > 30) in one. The analyses presented in Tables 1–5 were repeated with these 12 participants excluded and the results are presented in Supplementary Tables S1–S5. Excluding these participants had no significant effect on the results of the analysis. The median probability of prodromal Parkinson’s in the 12 participants with significant comorbid OSA was 99.0%.

### Conversion to Defined Neurodegenerative Disease

The mean duration of follow-up for the RBD cohort at the time of writing is 2.1 years (SD 1.25). Of the 171 patients with RBD recruited to the study, 16 have subsequently been diagnosed with a defined neurodegenerative disorder. The diagnosis was PD in nine participants; DLB in one; MSA in two; dementia without parkinsonism in three participants, and pure autonomic failure in one. Neurodegenerative diagnoses were made after a mean latency of 5.6 years (SD 2.45) from PSG confirmation of RBD and 9.8 years (SD 3.17) from RBD symptom onset. Three of the patients who converted to a neurodegenerative disorder had concomitant moderate OSA (AHI 15–30). Of the 10 patients who converted to PD or DLB, eight met the MDS criteria for probable prodromal Parkinson’s at baseline.

## DISCUSSION

In the largest study to date comparing the clinical phenotype of patients with RBD to that of patients with PD and healthy controls, we have demonstrated evidence of motor, autonomic, mood, and cognitive impairment in patients with RBD. In every nonmotor feature, participants with RBD are at least as impaired as patients with early PD, and in measures of depression, anxiety, and apathy, patients with RBD score worse than those with established Parkinson’s. This may explain the finding that patients with RBD rate their QOL as low as patients with early PD. We have previously shown that in patients with established PD, the presence of RBD is associated with lower QOL.^36^ Our data suggest that this effect appears during the prodromal phase and highlight the importance of recognizing and actively managing nonmotor symptoms.

The fact that patients with RBD do not exhibit an intermediate nonmotor phenotype between controls and patients with PD is in keeping with evidence that patients with PD who progress from idiopathic RBD tend to develop the akinetic-rigid/postural instability-gait difficulty subtype of disease,^1^ which is associated with a more severe nonmotor phenotype.^37^ This may also explain why our RBD and early patients with PD are equally impaired on the flamingo test (a measure of postural instability) and the get-up-and-go test (a measure of gait) despite patients with RBD having substantially lower UPDRS III scores.

Among demographic and environmental variables, we found that smoking and history of head injury were more common in patients with RBD than controls and duration of formal education was shorter, replicating the findings of a large multicentre study of risk factors for RBD.^38^ We also found that exposure to chemical solvents, but not pesticides, was more common in RBD and that the total number of cardiovascular risk factors was higher compared to controls. The findings with respect to head injury and solvent exposure are consistent with their known status as risk factors for Parkinson’s. The higher prevalence of smoking on the other hand is in conflict with the protective effect observed in relation to PD. The explanation for this is not clear; it remains uncertain whether there is indeed a real effect of smoking and vascular risk on the development of RBD or whether these differences are a result of selection bias in RBD cohorts.

Our findings support recent studies of the association between RBD and mutations in the LRRK2 and GBA genes. Taking our data alongside the only other published study of LRRK2 in idiopathic RBD,^17^ no mutations have been found in a combined total of 261 patients with RBD, substantially less than the prevalence of around 3% seen in sporadic PD.^39^ This provides further evidence that LRRK2-PD is associated with a lower incidence of RBD in the prodromal phase of the disease. In contrast, we found a higher prevalence of two common GBA mutations in RBD compared with controls. Although this result was of borderline statistical significance, it is in keeping with recent evidence linking GBA mutations with RBD in both PD and non-PD GBA carriers.^18,20,40^

We have shown that in patients with idiopathic RBD, lower BMI is associated with worse hyposmia, a common feature of prodromal PD corresponding to stage 1 of the Braak pathological staging system.^33^ Importantly, this difference is not seen in the control group, suggesting that the effect of BMI is specifically related to RBD. One possible explanation for this is the association between higher BMI and respiratory sleep disorders. OSA is a common condition that frequently coexists with RBD,^11–13^ and it is not uncommon for patients to be diagnosed with RBD following presentation to the sleep clinic with suspected OSA.^14^ We suggest that the presence of even mild OSA or other sleep-disordered breathing may prompt earlier referral to a sleep center and consequent diagnosis of RBD at an earlier prodromal stage than in those with RBD alone. Bias toward the referral of obese individuals may also underlie the significantly higher rate of obesity observed between RBD and control participants.

A similar effect is seen with antidepressant medications, use of which has been reported elsewhere as associated with a substantially reduced risk of conversion from RBD to neurodegenerative disease.^6^ Antidepressants are known to exacerbate RBD,^10^ and their use may therefore also lead to earlier PSG examination. In keeping with this hypothesis, patients with RBD taking antidepressants are younger and have less hyposmia and orthostatic hypotension than those not taking antidepressants, an effect that is not seen in the control group.

Combining these factors with age, we demonstrate that patients with RBD considered low risk in all three measures have an OR of 3.39 (95% CI 0.78–14.8) compared to controls for impaired olfaction, a difference that is consistent with chance. In those considered high risk in all three categories, the OR is 45.5 (95% CI 21.1–98). Longitudinal follow-up will establish whether these “high-risk” patients are more likely to convert to a neurodegenerative disorder, but the differences are striking and suggest that simple demographic data can contribute significantly to risk stratification.

The finding that 73.7% of patients with RBD fulfil the MDS criteria for probable prodromal PD is in line with longitudinal studies demonstrating a similar rate of conversion to neurodegenerative disease.^6^ However, this figure is largely accounted for by the PSG diagnosis of RBD itself, as excluding the likelihood ratio relating to this and using the RBDSQ instead would result in just 12% of patients with RBD fulfilling the criteria. Although this reflects the importance of PSG-confirmed RBD as a prodromal marker, it also highlights the reliance of these criteria on specialist investigations in order to obtain high sensitivity. This is further illustrated by the result in our early PD cohort, where patients did not undergo PSG or other invasive investigations. Using simple clinical measures only, just 21.3% of these patients met the criteria (NB although these patients by definition are not prodromal, the diagnosis of PD is recent [median time since diagnosis 0.56 years], and the risk factors incorporated in the MDS criteria will not reduce with time, so one can assume that probability scores would have been the same or lower in the prodromal phase). If, on the other hand, all these patients had had positive neuroimaging with DAT SPECT, the sensitivity of the MDS criteria would improve to 87%.

The low number of control participants fulfilling the criteria (0.3%) makes this a potentially useful tool for recruitment of prodromal patients to clinical trials, where a low false-positive rate may take precedence over high sensitivity. The sensitivity of the criteria could be increased by using a threshold of 50% instead of 80%, as others have suggested.^8^ In this instance, 98.6% of our healthy controls would still fall below the threshold, but 51.3% of patients with early PD would now meet criteria using simple clinical measures alone and 92.4% of participants with RBD. Importantly, 100% of the patients with RBD in our cohort who converted to PD or DLB would have met the 50% threshold at baseline.

Some limitations of this study should be noted. Interpretation of the demographic differences is somewhat limited by the fact that the RBD cohort was recruited from sleep centers throughout the United Kingdom, whereas the PD and control participants were recruited only from the Thames Valley region. Although our findings are consistent with two other large studies evaluating environmental risk factors for RBD,^38,41^ it is possible that in our study the differences simply reflect confounding by geographical variation. The large number of participants included in this study meant that it was not feasible to undertake PSG on the control or participants with PD. However, the primary comparison was between RBD and controls, and RBD is rare in the general population. If a small number of patients with RBD were inadvertently present in the control group, the effect would be small and would be likely to reduce the differences observed between RBD and control participants rather than exaggerate them.

In conclusion, our study has demonstrated extensive evidence of neurodegeneration in a large RBD cohort that is in keeping with the prodromal phase of alpha-synucleinopathies. Our data suggest that simple clinical measures can be used to risk stratify these patients, though this requires further replication. Longitudinal follow-up is underway and will establsh the true predictive value of these methods. Work to further refine the stratification model with novel neuroimaging and molecular tests is ongoing in our group. These tests will be important in selecting those at highest risk of conversion so that outcomes can be assessed within a time scale feasible for clinical trials of neuroprotective agents.

## FUNDING

This study was funded by the Monument Trust Discovery Award from Parkinson’s United Kingdom and supported by the National Institute for Health Research (NIHR) Oxford Biomedical Research Centre based at Oxford University Hospitals NHS Trust and University of Oxford, and the Dementias and Neurodegenerative Diseases Research Network (DeNDRoN). The views expressed are those of the author(s) and not necessarily those of the NHS, the NIHR, or the Department of Health.The funding agency had no role in the design and conduct of the study; collection, management, analysis, or interpretation of the data; preparation, review, or approval of the manuscript; or decision to submit the manuscript for publication.

## DISCLOSURE STATEMENT

TB and MR received funding from the National Institute for Health Research Biomedical Research Centre, based at Oxford University Hospitals Trust, Oxford. TB received salary from the Oxford Parkinson’s Disease Centre. ML was employed by the University of Bristol and funded by Parkinson’s United Kingdom. FB, SE, and CR were employed by the Oxford Parkinson’s Disease Centre, Nuffield Dept of Clinical Neurosciences, University of Oxford. AG is funded by the National Institute for Health Research (NIHR) Oxford Biomedical Research Centre based at Oxford University Hospitals NHS Trust and University of Oxford. CL was funded by the National Institute for Health Research. GD is employed by Sheffield University Hospitals NHS Foundation Trust. OB is employed by the University of Sheffield and received research funding from Parkinson’s United Kingdom and the Michael J Fox Foundation. OB received speaker honoraria from UCB. TQ was employed by Papworth Hospital NHS Foundation Trust. ZZ was employed by Oxford University Hospitals NHS Trust. YB-S was employed by the University of Bristol and received research funding from Parkinson’s United Kingdom, ESRC/NIA, National Institute of Health Research, Medical Research Council and the Brain Tumour Charity. YB-S is a member of the Multiple Sclerosis risk-sharing scheme scientific advisory board and received royalties from Oxford University Press and Wiley. MH is funded by the OPDC Monument Discovery award, the Oxford Biomedical Research Centre and National Institute of Health Research Clinical Research Network.

## SUPPLEMENTARY MATERIAL

Supplementary material is available at *SLEEP* online.

## Supplementary Material

Supplementary_Tables_S1-S5
