# Supplementary material for: Prodromal Parkinsonism and Neurodegenerative Risk Stratification in REM Sleep Behavior Disorder
Source: Sleep. 2017 May 4;40(8):zsx071. doi: 10.1093/sleep/zsx071 (PMC5806544; doi:10.1093/sleep/zsx071)
Supplement: Supplementary_Tables_S1-S5 [file zsx071_suppl_supplementary_tables_s1-s5.docx]

Table S1. Demographics and background risk factors

|  | Controls  N=296 | RBD  N=159 | PD  N=119 |  | P value |
| --- | --- | --- | --- | --- | --- |
| Age  Mean (SD) | 64.9 (10.2) | 64.8 (8.7) | 66.9 (9.1) | RBD vs Control:  RBD vs PD:  PD vs Control: | 0.84  0.07  0.06 |
| Sex  (% male) | 49.0 | 88.7 | 70.6 | RBD vs Control:  RBD vs PD:  PD vs Control: | **<0.001**  **<0.001**  **<0.001** |
| BMI  Mean, kg/m^2^ (SD) | 27.4 (4.93) | 28.9 (5.83) | 26.1 (3.73) | RBD vs Control:  RBD vs PD:  PD vs Control: | **0.009**  **<0.001**  **0.02** |
| Pesticide Exposure* (%) | 37.2 | 45.3 | 33.9 | RBD vs Control:  RBD vs PD:  PD vs Control: | 0.17  0.07  0.44 |
| Solvent Exposure+ (%) | 11.1 | 22.0 | 18.6 | RBD vs Control:  RBD vs PD:  PD vs Control: | 0.06  0.95  0.10 |
| Caffeine Intake** | 4.71 (2.24) | 5.27 (2.67) | 5.00 (2.87) | RBD vs Control:  RBD vs PD:  PD vs Control: | 0.19  0.63  0.47 |
| Head Injury++ (%) | 18.2 | 32.1 | 22.9 | RBD vs Control:  RBD vs PD:  PD vs Control: | **0.02**  0.24  0.41 |
| Smoking History*** (%) | 43.2 | 63.5 | 40.2 | RBD vs Control:  RBD vs PD:  PD vs Control: | **0.002**  **<0.001**  0.31 |
| Obesity^ (%) | 23.3 | 35.8 | 16.0 | RBD vs Control:  RBD vs PD:  PD vs Control: | **0.004**  **<0.001**  0.16 |
| Education (yrs) | 15.1 (3.45) | 13.8 (3.42) | 15.1 (3.86) | RBD vs Control:  RBD vs PD:  PD vs Control: | **<0.001**  **<0.001**  0.64 |
| Antidepressant use (%) | 11.5 | 29.6 | 12.6 | RBD vs Control:  RBD vs PD:  PD vs Control: | **<0.001**  **<0.001**  0.25 |
| GBA Mutation (%) | 0.40 | 2.8 | 0.90 | RBD vs Control:  RBD vs PD:  PD vs Control: | **0.05**  0.28  0.40 |
| LRRK2 Mutation (%) | 0 | 0 | 0.90 | RBD vs Control:  RBD vs PD:  PD vs Control: | n/a  1.0  0.99 |
| Total number of cardiovascular risk factors, Mean (SD)+++ | 1.01 (1.11) | 1.58 (1.45) | 1.08 (1.14) | RBD vs Control:  RBD vs PD:  PD vs Control: | **<0.001**  **<0.001**  0.98 |

*Exposure to pesticides at work or home; +Exposure to chemical solvents for >6 months; **Past caffeine intake, number of caffeinated drinks per day; ++History of head injury causing loss of consciousness or concussion diagnosed by a doctor; ***Past or current smoking history; ^BMI >30 Kg/m^2^; +++risk factors defined as: history of cardiovascular disease (angina, myocardial infarction, stroke or transient ischaemic attack), diabetes, obesity, hypertension, current smoker, hypercholesterolaemia. For all variables except age, sex and GBA/LRRK2 status, p-values for between groups comparisons are corrected for age and sex. The comparison of GBA/LRRK2 status is adjusted for sex only.

Table S2. Motor and non-motor features

|  | Controls  N = 296 | RBD  N = 159 | PD  N = 119 | Significance  (p values) | |
| --- | --- | --- | --- | --- | --- |
| Motor | Values are mean (SD) unless otherwise stated | | |  | |
| UPDRS III, score | 1.74 (2.74) | 4.47 (4.13) | 25.7 (11.1) | RBD vs Controls:  RBD vs PD:  PD vs Controls: | **<0.001**  **<0.001**  **<0.001** |
| Purdue pegboard, score | 37.5 (6.80) | 37.2 (7.93) | 28.6 (6.49) | RBD vs Controls:  RBD vs PD:  PD vs Controls: | 0.26  **<0.001**  **<0.001** |
| Flamingo, % | 71.0 | 55.5 | 55.3 | RBD vs Controls:  RBD vs PD:  PD vs Controls: | **<0.001**  0.23  **0.002** |
| Get up and go, time | 8.51 (1.73) | 9.15 (2.42) | 9.56 (2.24) | RBD vs Controls:  RBD vs PD:  PD vs Controls: | **<0.001**  0.38  **<0.001** |
| Non-Motor |  |  |  |  | |
| MMSE, score | 28.3 (1.89) | 27.3 (2.08) | 27.6 (2.29) | RBD vs Controls:  RBD vs PD:  PD vs Controls: | **<0.001**  0.111  **0.002** |
| MoCA, score | 26.7 (2.67) | 25.2 (2.78) | 25.2 (3.32) | RBD vs Controls:  RBD vs PD:  PD vs Controls: | **<0.001**  0.85  **<0.001** |
| Mild Cognitive Impairment (MoCA <24) % | 12.5 | 24.1 | 27.6 | RBD vs Controls:  RBD vs PD:  PD vs Controls: | **0.02**  0.51  **0.003** |
| Semantic fluency, score | 12.0 (3.33) | 9.85 (3.38) | 10.5 (3.34) | RBD vs Controls:  RBD vs PD:  PD vs Controls: | **<0.001**  0.18  **<0.001** |
| Phonemic fluency, score | 12.8 (3.72) | 10.5 (4.07) | 11.5 (4.01) | RBD vs Controls:  RBD vs PD:  PD vs Controls: | **<0.001**  0.06  **0.004** |
| Sniffin’ sticks, score | 12.1 (2.28) | 8.14 (3.26) | 7.46 (2.88) | RBD vs Controls:  RBD vs PD:  PD vs Controls: | **<0.001**  **0.05**  **<0.001** |
| Orthostatic systolic blood pressure drop, mmHg | 0.09 (12.3) | 5.47 (13.6) | 3.75 (13.5) | RBD vs Controls:  RBD vs PD:  PD vs Controls: | **<0.001**  0.26  **0.031** |
| Postural Lightheadedness % | 12.2* | 39.7 | 27.1 | RBD vs Controls:  RBD vs PD:  PD vs Controls: | **<0.001**  **0.03**  **0.005** |
| Constipation, % | 34.7 | 48.4 | 39.8 | RBD vs Controls:  RBD vs PD:  PD vs Controls: | **0.001**  **0.05**  0.34 |
| Beck Depression Inventory Score | 4.85 (5.02) | 9.46 (8.90) | 7.55 (5.88) | RBD vs Controls:  RBD vs PD:  PD vs Controls: | **<0.001**  **0.02**  **<0.001** |
| Leeds Anxiety Score | 2.12 (2.38) | 4.10 (3.72) | 2.77 (2.98) | RBD vs Controls:  RBD vs PD:  PD vs Controls: | **<0.001**  **<0.001**  **0.007** |
| Apathy (UPDRS part I, % >0) | Not measured | 28.9 | 16.2 | RBD vs PD | **0.02** |
| Quality of Life  EQ5D % score | 84.5 (10.6) | 74.4 (19.6) | 76.0 (14.4) | RBD vs Controls:  RBD vs PD:  PD vs Controls: | **<0.001**  0.40  **<0.001** |
| UPDRS I, total score | Not measured | 9.11 (6.41) | 6.95 (4.53) | RBD vs PD | **0.001** |

All p-values for two-way comparisons are corrected for age and gender differences between the groups. *data regarding this symptom was only available from 114 controls

Table S3. Increased risk of parkinsonian features in RBD vs Controls:

|  | RBD vs Controls adjusted for age and gender  Odds ratio (95% CI) |
| --- | --- |
| Motor impairment^ | 6.50 (3.64 – 11.6) |
| Cognitive impairment* | 1.99 (1.15 – 3.47) |
| Hyposmia** | 13.3 (7.79 – 22.8) |
| Depression*** | 6.16 (3.11 – 12.18) |
| Anxiety**** | 5.85 (2.71 – 12.6) |
| Constipation+ | 2.21 (1.41 – 3.46) |
| Orthostatic hypotension++ | 3.99 (1.92 – 8.26) |

Defined as: ^UPDRS III score >4; *MoCA <24; **Sniffin’ Sticks<10; ***BDI-II >13; ****Leeds Anxiety Score >6; +less than 1 bowel movement per day or use of laxatives; ++Orthostatic drop in systolic blood pressure >20 mmHg

Table S4. RBD patient stratification and risk of hyposmia

| ***Not on antidepressants*** | ***BMI < 30*** | ***Age > 60*** | ***Odds ratio (95 % CI) for hyposmia*, RBD vs. Control*** |
| --- | --- | --- | --- |
| No | No | No | 2.85 (0.62, 13.1) |
| Yes | No | No | 5.24 (1.43, 19.3) |
| No | No | Yes | 5.96 (1.50, 23.6) |
| Yes | No | Yes | 11.0 (3.96, 30.4) |
| No | Yes | No | 11.8 (2.39, 57.8) |
| Yes | Yes | No | 21.7 (6.98, 67.1) |
| No | Yes | Yes | 24.6 (5.79, 104.5) |
| Yes | Yes | Yes | 45.3 (20.6, 99.5) |

*hyposmia defined as Sniffin Sticks score < 10^th^ centile of normative data adjusted for age and gender

Table S5. MDS Criteria for Prodromal Parkinson’s according to PD, RBD or control status at baseline and for RBD pheno-convertors to PD/DLB

|  | Controls  n=296 | RBD  N=159 | PD  N=119 | Converted from RBD to PD or DLB at follow up  (N=8) |
| --- | --- | --- | --- | --- |
| Observed median probability of prodromal PD | 0.48% | 92.3% | 52.2% | 94.4% |
| >80% probability | 0.3% | 73.6% | 21.8% | 75.0% |
| >50% probability | 1.40% | 92.5% | 51.3% | 100% |

All values are at baseline evaluation
